# The Effects of Motor–Cognitive Warm-Up Protocols on Sport-Specific Skills in 8-Year-Old Football Players

**DOI:** 10.3390/sports13120416

**Published:** 2025-11-25

**Authors:** Sayyedarmin Ganji, Hamidreza Sepehri Rahnama, Sára Németh, Dominika Jantal, Kitty Vadasz, Judit Prokai

**Affiliations:** Institute of Sport Sciences and Physical Education, Faculty of Sciences, University of Pécs, 7624 Pécs, Hungary; hamidrezasepehri01@gmail.com (H.S.R.); nemeth.sara99@gmail.com (S.N.); dominikajantal@gmail.com (D.J.); vadaszki@gamma.ttk.pte.hu (K.V.); prokai@gamma.ttk.pte.hu (J.P.)

**Keywords:** motor-cognitive warm-up, auditory stimuli, visual stimuli, executive functions, working memory, agility, youth football, passing accuracy, stroop test, cognitive performance

## Abstract

Football is a multifaceted sport in which cognitive function plays a crucial role alongside physical performance in overall athletic success. However, widely adopted warm-up protocols primarily target motor skills, with minimal attention to cognitive readiness. This study aimed to investigate the short-term effects of motor–cognitive warm-up protocols incorporating auditory and visual stimuli on various cognitive and motor skills in youth male football players. Twenty-four male players (age = 8.56 ± 0.33 years) were randomly assigned to three groups (*n* = 8): motor–verbal (MVEG), motor–visual (MVIG), and motor-only. Each player completed one warm-up session followed by four performance tests. All groups completed the same football-specific warm-up exercises, differing only in instruction modality: pre-given for MG, verbal (auditory) for MVEG, and visual for MVIG. Immediately after the warm-up, participants completed the Stroop Color-Word Test (SCWT), Loughborough Soccer Passing Test (LSPT), Illinois Ball Test (IBT), and *t*-test. Significant group differences were found in SCWT error rate (*p* = 0.009), LSPT time (*p* = 0.001), IBT time (*p* = 0.036), and *t*-test time (*p* = 0.003). Across these tests, seven outcome measures were recorded: SCWT completion time and error rate, LSPT total time (finishing time plus penalties), IBT completion time and error rate, and *t*-test completion time. No differences were observed in SCWT completion time or IBT error rate. MVEG outperformed the other groups in most tests, except the LSPT, where MVIG achieved the best performance. MG showed the lowest overall performance, except in the *t*-test. Warm-up protocols incorporating cognitive stimuli can immediately improve motor and executive performance.

## 1. Introduction

Football requires players to make rapid decisions, maintain spatial awareness, and seize dynamic opportunities [1]. They must quickly extract relevant information through flexible visual exploration strategies and brief fixation on informative cues [2], plan appropriate responses, and anticipate their opponents’ future actions [3]. These demands engage a broad spectrum of cognitive processes, making higher cognitive function (CF) levels essential for success.

CF encompasses a range of mental abilities, including attention, perception, visual and spatial processing, and executive functions (EF), such as decision-making, working memory, and multi-tasking [4]. Additional core EF components include updating, shifting, and inhibition [5] and cognitive flexibility [6]. These abilities are crucial for executing rapid and appropriate responses, especially under time pressure and uncertainty. Accordingly, football performance often hinges on the ability to respond effectively to unpredictable game situations and anticipate opponents’ strategies [3]. Research suggests that higher EF correlates with better performance outcomes, including increased goals and assists [3].

Long-term interventions have shown that EF can be improved through both physical activities (e.g., aerobic exercise) and cognitive training (e.g., mental arithmetic). Notably, combining these in motor–cognitive dual-task training appears more beneficial, as it recruits more mental resources and enhances both shifting ability and working memory [5]. For example, in adolescent shooting athletes, combined physical and cognitive exercises improved EF and brain oxygenation more than cognitive training alone [7]. Moreover, interactions between cognitive and motor skills influence the functional and structural development of brain networks [8].

The significance of CF in football is well documented [9], with several studies highlighting that CF performance can be just as critical as physical performance for overall success [10]. Football itself supports the development of cognitive abilities [11], yet strategies for systematically improving CF in young players remain underexplored. Since children around the age of ten are undergoing biological maturation of EF, introducing motor–cognitive training during this sensitive developmental period may further support both neural development and athletic performance. Childhood is especially critical, as key regions such as the prefrontal cortex and limbic system are actively developing, forming the foundation for executive functions [12].

Most existing studies focus on long-term interventions or laboratory-based cognitive tasks, rather than evaluating the short-term effects of CF enhancement in real football settings. Warm-ups may present a promising context for such interventions, as they activate EF processes [8,13]. A recent study compared two warm-up protocols: a small-sided 5 v 5 game (integrated) versus twelve dynamic, football-specific exercises (analytical). The integrated protocol led to significantly better technical–decisional performance, as measured by the Loughborough Soccer Passing Test (LSPT) [14]. However, that study did not directly assess executive functions, the role of sensory stimuli, or the design of a structured motor–cognitive warm-up.

Our study addresses these gaps by introducing visual and auditory stimuli within warm-up routines and investigating their short-term effects on executive functions, decision-making, and motor–cognitive integration during football-specific tasks. Previous methodologies have often overlooked the reality that footballers operate in environments heavily influenced by visual and verbal cues. With over 90% of information acquired through visual and auditory channels, these modalities are vital for the cognitive process [15]. For instance, visual exploration frequency correlates with pass completion rates [16], while auditory cues help athletes distinguish their own movements from those of opponents, aiding environmental perception and action prediction [17].

In light of this, incorporating targeted cognitive drills into football warm-ups may enhance mental readiness and performance. To our knowledge, no previous study has systematically examined the effects of football-specific, motor–cognitive warm-up tasks using visual and auditory cues. Therefore, this study aims to investigate their short-term effects on motor and cognitive performance in youth male football players.

## 2. Materials and Methods

### 2.1. Participants

Twenty-four youth male football players (*n* = 24, age = 8.56 ± 0.33 years, weight = 30.08 ± 4.76 kg, height = 133.10 ± 4.66 cm, BMI = 16.85 ± 1.77) were recruited for this study. Parental consent was obtained through signed informed declarations. Players with current or recent injuries, or known motor/cognitive special skills were excluded. All remaining participants were healthy and injury-free at the time of testing. The players had trained together for two years on the same team under identical training conditions. Due to their age, they had no fixed playing positions and rotated across roles during training sessions and matches. Before testing, participants attended a familiarization session. To prevent cross-group observation and potential confusion, each group was isolated from the others. A trained assistant provided a comprehensive explanation of the designated warm-up and testing procedures, followed by a live demonstration. Notably, participants did not actively perform the tasks during familiarization to minimize potential learning effects and practice-related performance gains. Participants observed the tasks (SCWT, LSPT, IBT, and *t*-test) and were encouraged to ask questions to ensure full understanding. An information sheet was provided to parents, players, and the club stating that participation was voluntary and withdrawal was permitted in the case of discomfort. The research was conducted on a natural grass pitch. The study was conducted in accordance with the Declaration of Helsinki. Approval for the publication of this article was provided by the Regional and Institutional Research Ethics Committee of the Medical School, Clinical Center, University of Pécs on the meeting held on the 12 September 2025 as the data connected to this article are processed ethically anonymously for scientific purpose (10198-PTE2025).

### 2.2. Experimental Protocol and Procedure

Participants were randomly assigned to one of three groups. All testing was conducted in a single session per participant, immediately following the warm-up protocol.

All groups performed different warm-up protocols, followed by standardized assessment using four tests: the Stroop Color-Word Test (SCWT), Loughborough Soccer Passing Test (LSPT), Illinois Ball Test (IBT), and *t*-test. A familiarization session preceded testing to ensure procedural clarity. Each participant completed the tests immediately following their warm-up. Test order and instructions were standardized and verbally explained by the examiner before each trial. The same examiner and assistants were involved throughout to eliminate inter-experimenter variability.

#### 2.2.1. Stroop Color-Word Test (SCWT)

**Figure 1:**
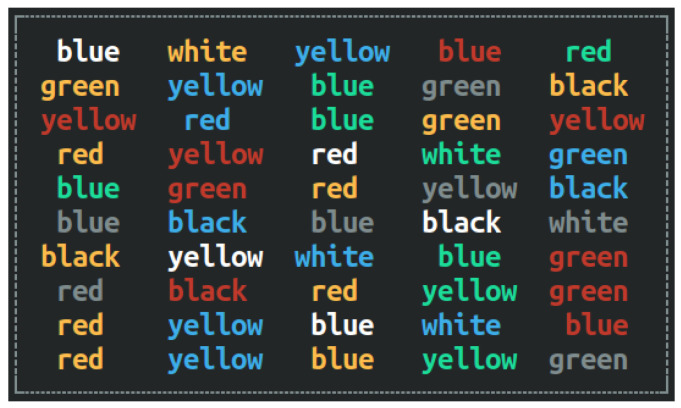
Schematic illustration of the Stroop Color-Word Test with incongruent stimuli.

SCWT is a validated computerized tool used to evaluate cognitive domains [18] such as inhibition, working memory, attention, and cognitive flexibility [19]. It is used to assess executive functions, requiring participants to inhibit their normal response in order to react to an unexpected choice [20]. The incongruent version was selected for its sensitivity to cognitive interference, where greater neural effort is required to suppress automatic responses [21]. This version involved ten trials aimed at enhancing mental readiness through color-naming interference, where Hungarian color words were displayed in mismatched font colors. For example, the word “piros” (red) appears in blue, requiring participants to select blue from three choices as quickly as possible. The test was administered via the iOS version of the Stroop app on an iPhone 15 Pro. Participants were instructed to respond solely based on font color, not the word itself, thus eliciting the classic “Stroop effect”, linked to prefrontal cortex activation [22]. Performance was evaluated based on total response time (in seconds) to trials, and if any participant was unable to choose the correct option, his response was counted as the number of errors (incorrect color-word responses), which was expressed as a percentage of total possible correct answers. Errors were not penalized through added time; they were analyzed separately (Figure 1).

#### 2.2.2. Loughborough Soccer Passing Test (LSPT)

**Figure 2:**
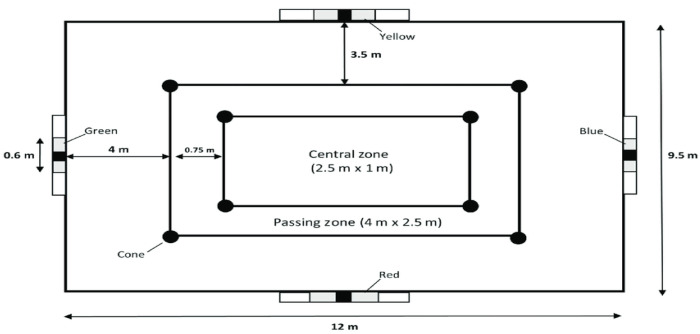
The schematic layout of the LSPT testing area.

LSPT assesses football-specific skills, including agility, passing accuracy, ball control, and decision-making, and cognitive abilities such as multi-tasking and attention, as players must recall target sequences and adapt their passes under time pressure [14,23,24]. Participants stood in a central zone surrounded by four colored target zones. At the examiner’s verbal cue, they passed the ball toward the named target as quickly and accurately as possible. Of the sixteen passes, eight were shorter (to yellow and red targets), and eight were longer (to green and blue targets), which the examiner called randomly for each participant. After each return, the next command was issued immediately. Performance was timed from start to finish, which means the participants had to complete the sixteen passes as fast as they could in order to submit a better result Time penalties were added to the finishing time of the test for the following: missing the target (three seconds), wrong target (five seconds), touching any cone with the ball or passing from outside the designated zone (two seconds), and exceeding the 43 s standard test duration (one second per second), The final score was the sum of the raw completion time plus penalty time, producing a single performance index where lower scores indicated better performance (Figure 2).

#### 2.2.3. Illinois Ball Test (IBT)

**Figure 3:**
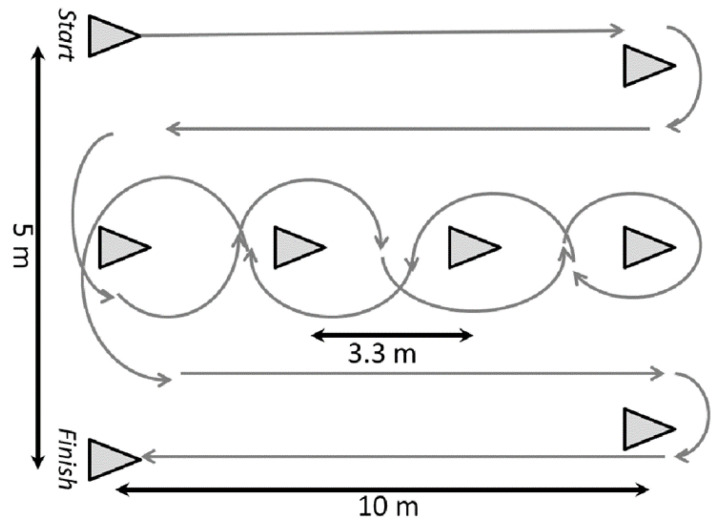
Schematic representation of the Illinois Ball Test.

The IBT evaluates agility with ball control and has demonstrated strong validity for youth football players [25]. Beyond physical agility, it engages multi-tasking skills, attentional focus, and decision-making processes—core cognitive demands reflective of real-game situations [26,27,28]. The course featured cones arranged to test quick directional changes and slalom dribbling at varied angles. At the whistle, players sprinted with the ball through the sequence. Performance was assessed based on two indicators: the total completion time of the test (in seconds) and the number of errors that they made. Errors included missing or skipping cones, dribbling in the wrong order, or losing control of the ball. Time and number of errors were recorded separately for performance analysis. (Figure 3).

#### 2.2.4. t-Test

**Figure 4:**
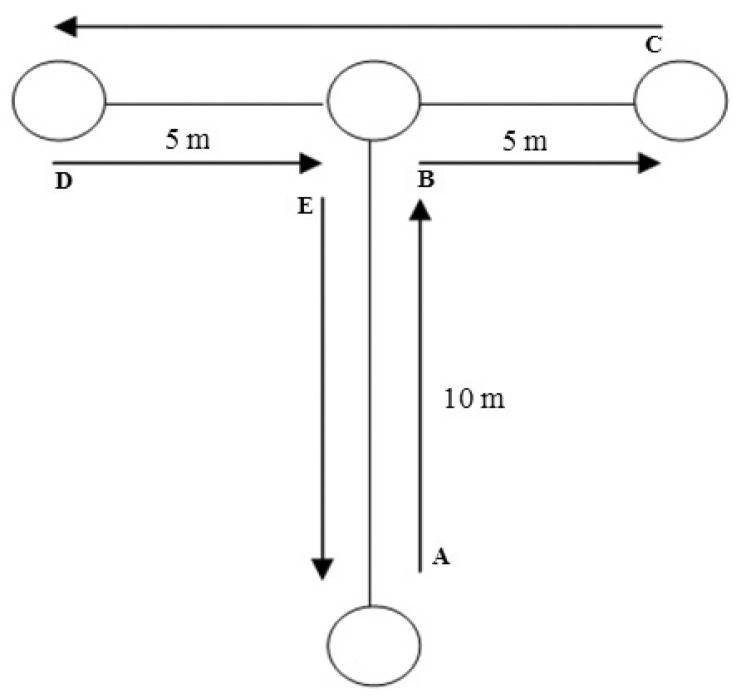
The schematic diagram of the *t*-test layout.

The *t*-test is a widely used measure of multi-directional agility, lower-body strength, and speed in athletes [26,27,28]. Unlike the IBT, it assesses agility without the ball. On the examiners’ whistle, participants sprinted forward, shuffled left, shuffled right, returned to the center, then backpedaled to the starting points [13]. Performance was recorded as total completion time (in seconds). To ensure valid execution, players were reminded of the correct technique (e.g., no crossing legs during shuffles, touching all required lines) before starting the test. For each error registered, one second was added to the total completion time, and the adjusted final score was used for analysis (Figure 4).

#### 2.2.5. Test Order

All participants completed the tests in the following sequence: Stroop Color-Word Test (SCWT), Loughborough Soccer Passing Test (LSPT), Illinois Ball Test (IBT), and *t*-test. This order was applied consistently across all groups to ensure standardization of the testing procedure. Adequate one-minute and thirty-second rest intervals were provided between tests to reduce fatigue carryover.

### 2.3. Warm-Up Protocol

Given the essential role of visual and auditory stimuli in sports performance, we supplemented motor tasks with sensory cues to examine their impact on motor–cognitive processing. Participants were randomly assigned to one of three groups (*n* = 8 per group): motor–verbal group (MVEG), motor–visual group (MVIG), and motor-only group (MG), which served as the control group. To prevent cross-group awareness, participants were positioned in separate areas during both the instructional and demonstration phases. Each individual received information solely about their designated warm-up protocol, ensuring they remained unaware of the procedures assigned to other groups. All groups performed the same motor tasks during their pre-training warm-up. However, the source and timing of instructional information differed. MG received complete instructions in advance, including the task content, order of actions, and movement direction (e.g., which way to shuffle, pass, or shoot). MVEG received task descriptions before the warm-up, but directional details were delivered verbally (e.g., “left,” “right,” “red,” or “green”) during execution. MVIG received the same delayed instructions, but via visual cues, including colored cards or hand signs. Each task lasted two minutes, except for the running exercise, which was performed individually for one minute. The exercises are described below.

**Figure 5:**
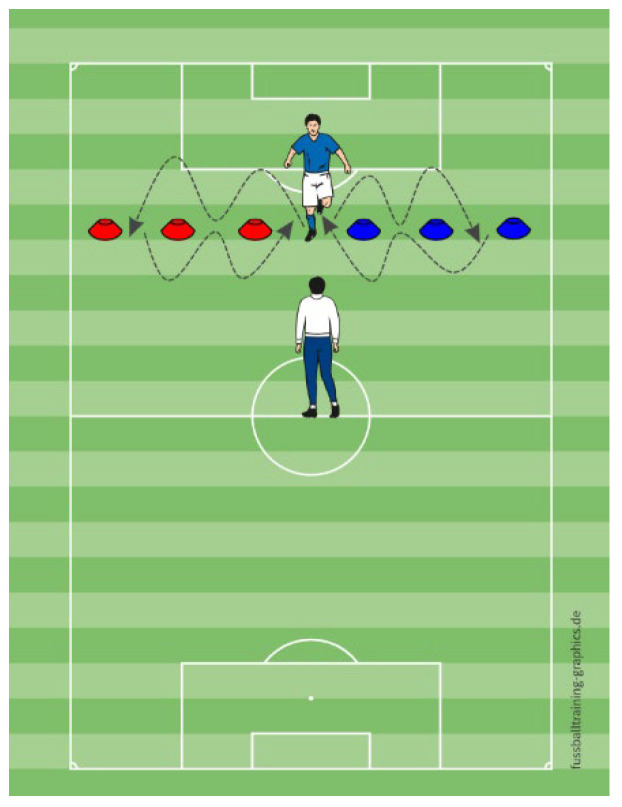
The schematic representation of the running exercise.

**Figure 6:**
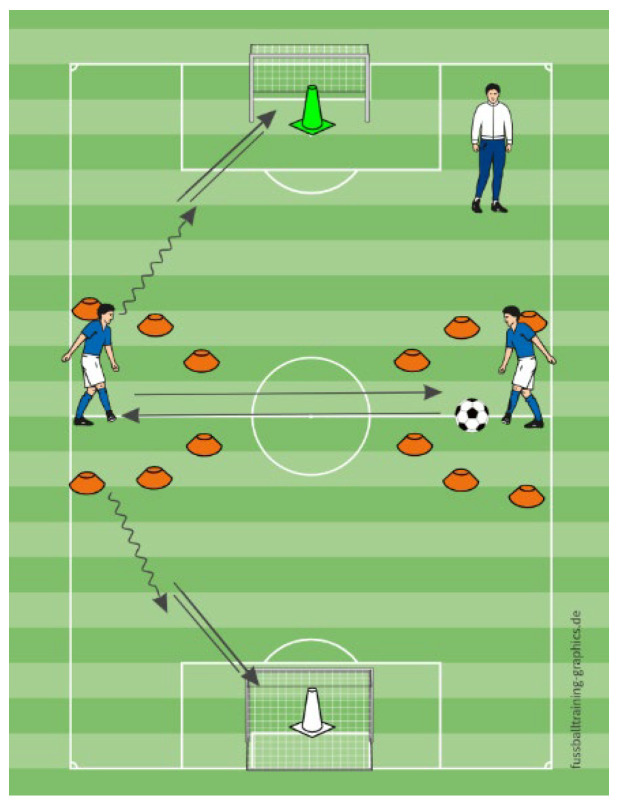
The schematic representation of the shooting exercise.

In the running exercise, participants laterally shuffled between cones positioned to their left and right, returning to a central start point each time (Figure 5). MG was pre-informed of the movement sequence (right–center–left–center). During the exercise, participants of MVEG responded to verbal instructions provided by the examiner, who vocally indicated the direction in which the player should shuffle. Upon returning to the center position, the examiner announced the subsequent directional cue. In contrast, the MVIG followed non-verbal hand signals that indicated the shuffle direction in the same sequence as MVEG, but without any spoken commands—only visual gestures pointing toward the intended direction. In the shooting exercise, participants and a helper passed the ball constantly in a confined area. At a designated time point, the participant who had the ball had to carry the ball outside the zone and shoot at a specific goal (right or left), alternating sides throughout the task. MG knew the shooting sequence and timing (after 10 passes) in advance, while MVEG relied on verbal commands that were pointing the goal that the player had to shoot the ball towards it (“LEFT” or “RIGHT”), to initiate the shot. MVIG responded to color cards (e.g., green or white) indicating the target goal which had a big colored cone in side of it, so the player could recognize the color that he had to shoot towards (Figure 6).

**Figure 7:**
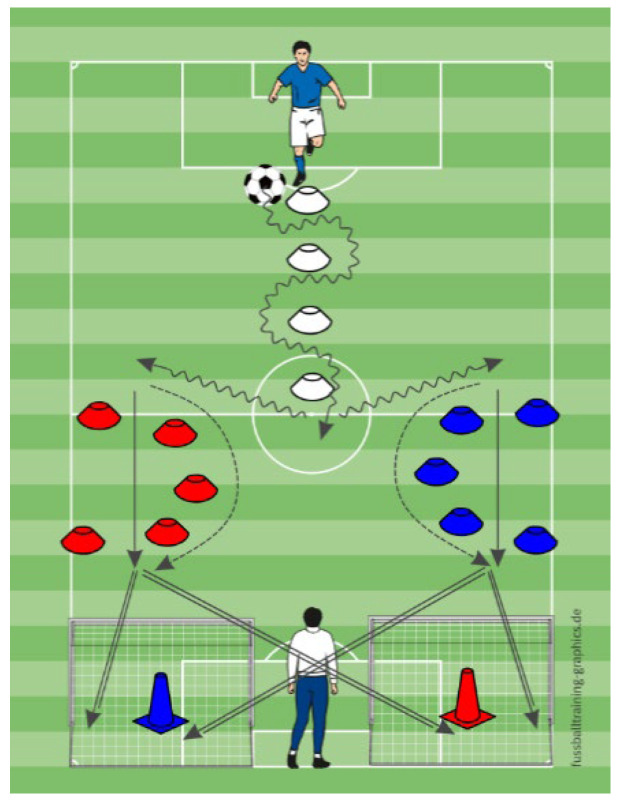
The schematic representation of the passing exercise.

In the passing exercise, four helpers stood at fixed positions near colored cones around the participant. The participant passed to the indicated helper and received the ball back (Figure 7). MG passed in a clockwise sequence with prior instruction. MVEG responded to verbal cues denoting cone color (e.g., “blue”). MVIG received visual prompts (colored cards) to identify the correct passing direction.

Explanation of schematic arrows:

~~~~> → Dribbling with the ball (Moving with the ball).

→ → Passing the ball.

⇒// → Shooting at goal (a double arrow is used to illustrate the action of finishing).

⋯⋯→ → Running without the ball (movement off the ball).

**Figure 8:**
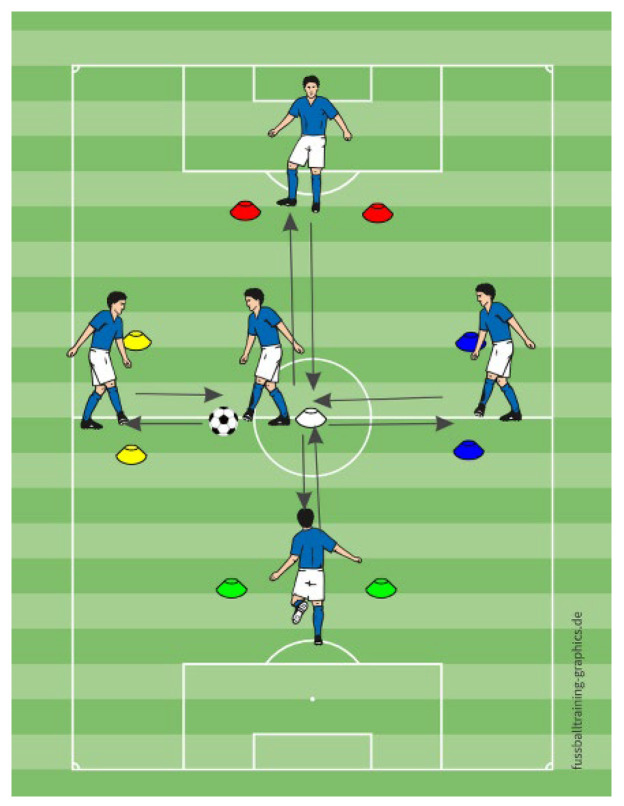
The schematic representation of dribbling exercise.

In the dribbling exercise, participants begin by slalom dribbling through a cone pattern. They then proceeded to the left or right side to complete a second dribbling sequence using a different technique (pass through the cones, and run around the zone to control the ball), followed by a shot into a small goal (Figure 8). MG received complete instructions in advance regarding direction, cone color, and goal target, so they knew in advance which cones they should go for the second part and which goal they need to shoot to at the end. MVEG was verbally cued about the dribbling cone color (e.g., “red” or “blue”) and shooting target (e.g., “red” or “blue”). MVIG followed the corresponding colored cards for both dribbling and shooting instructions.

Each warm-up exercise was continuously performed for two minutes by every participant under the guidance of a licensed instructor. Warm-ups were conducted in separate areas according to group allocation and followed standardized instructions. Participants were instructed to maintain a consistent level of effort, emphasizing accuracy and adherence to the prescribed sequence. 

### 2.4. Statistical Analyses

All statistical analyses were performed using OriginLab 2018 (OriginLab Corporation, Northampton, MA, USA). Descriptive statistics (mean ± standard deviation) were computed for all measured and calculated variables. Prior to hypothesis testing, data were assessed for normality using the Shapiro–Wilk test. To examine the short-term effects of the different warm-up protocols on motor and cognitive performance, a one-way ANOVA test was conducted to compare post-intervention results among the three groups (MVEG, MVIG, and MG) for each test (SCWT, LSPT, IBT, and *t*-test). Following significant ANOVA results, pairwise independent-sample *t*-test with Bonferroni correction was used to identify between-group differences. The significance level was set at *p* < 0.05. Effect sizes for ANOVA results were defined as small (η^2^ ≤ 0.01), medium (0.01 < η^2^ > 0.06), and large (η^2^ ≥ 0.14). SPSS 28 (SPSS Inc., IBM, Chicago, IL, USA) was used to carry out the statistical analysis [23].

## 3. Results

Twenty-four participants were randomly divided into three groups. Based on baseline anthropometric characteristics and training experience, there were no statistically significant differences between groups (Table 1).

### 3.1. Stroop Color-Word Test

**Figure 9:**
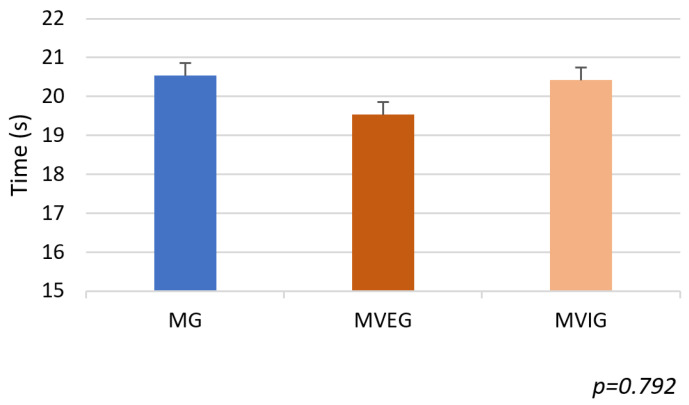
The time taken to complete SCWT.

**Figure 10:**
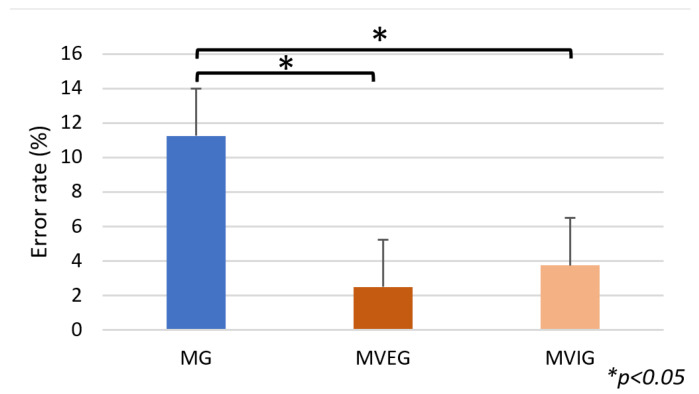
The sum of the errors in percentage in the Stroop Color-Word Test based on the wrong choices of participants.

There were no significant differences among groups in total completion time of the SCWT (F(2,21) = 0.24, *p* = 0.79, η^2^ = 0.02) (Figure 9). However, a significant difference was observed in the error rates for incongruent trials (F(2,21) = 6.02, *p* = 0.009, η^2^ = 0.36). Both MVEG (2.5 ± 4.63%) and MVIG (3.75 ± 5.18%) made fewer errors than MG (11.25 ± 6.41%), with MVEG showing the lowest error rate overall (Figure 10).

### 3.2. Loughborough Soccer Passing Test

**Figure 11:**
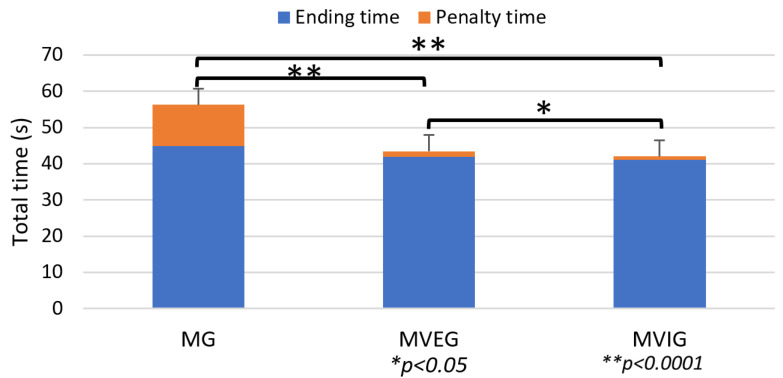
Total time of LSPT, including finishing and penalty time.

A significant difference in total test time (including penalties) was found across groups (F(2,21) = 11.02, *p* = 0.001, η^2^ = 0.51). Both MVEG (43.45 ± 6.04 s) and MVIG (41.97 ± 4.21 s) outperformed MG (56.22 ± 8.07 s), with MVIG achieving the best overall performance, having the shortest completion time and fewest penalty seconds (Figure 11).

### 3.3. Illinois Ball Test

**Figure 12:**
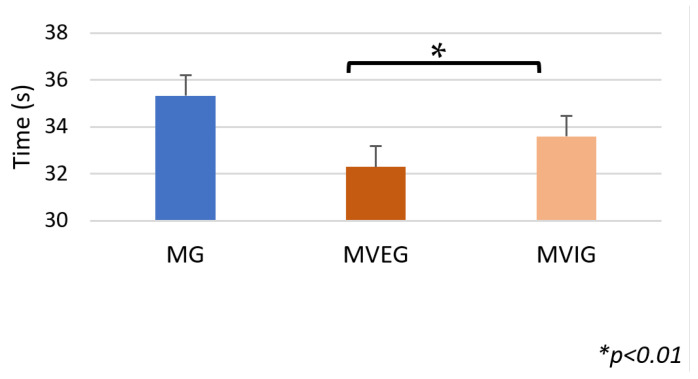
The time taken to complete the IBT.

**Figure 13:**
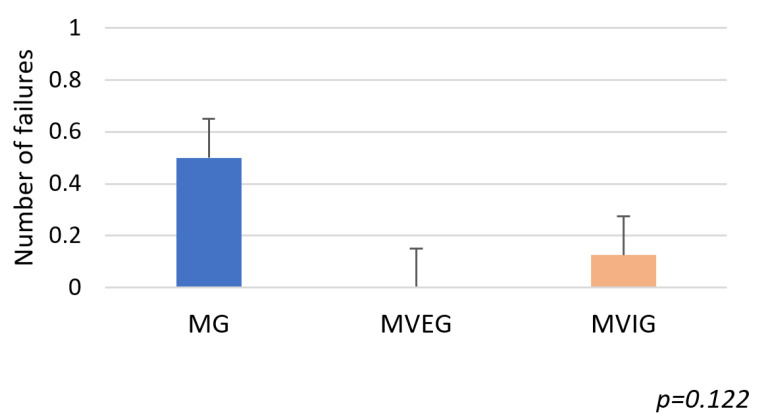
The number of failures in the Illinois Ball Test.

Time to complete the IBT differed significantly between groups (F(2,21) = 3.85, *p* = 0.036, η^2^ = 0.27). MVEG (32.29 ± 1.96 s) and MVIG (33.59 ± 2.02 s) were both faster than MG (35.33 ± 2.56 s), with MVEG again outperforming MVIG. (Figure 12). The number of failures did not show any significance (F(2,21) = 2.33, *p* = 0.12, η^2^ = 0.18); however, MVEG performed with zero errors (0 ± 0), followed by MVIG (0.13 ± 0.35), while MG (0.5 ± 0.76) had the highest number of errors (Figure 13).

### 3.4. t-Test

**Figure 14:**
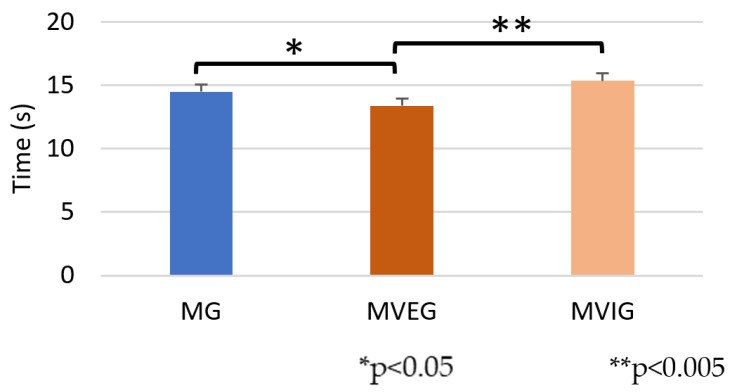
The time taken to complete the *t*-test.

Significant group differences were observed in *t*-test performance times (F(2,21) = 6.02, *p* = 0.003, η^2^ = 0.36). MVEG achieved the fastest completion time (13.38 ± 1.02 s), followed by MG (14.49 ± 0.87 s), and MVIG had the longest time (15.36 ± 1.08) (Figure 14).

## 4. Discussion

This study investigated the short-term effects of three warm-up protocols—motor-only (MG), motor–verbal (MVEG), and motor–visual (MVIG)—on cognitive and motor performance in youth male football players. The main findings were as follows: (i) significant group differences in SCWT error rate, LSPT, IBT time, and *t*-test performance; (ii) no significant differences in SCWT completion time or IBT error rate; (iii) overall superior performance by MVEG, with MVIG also outperforming MG in most tasks, except the *t*-test, where MG ranked second; MVIG achieved the best outcomes in the LSPT.

Both MVEG and MVIG demonstrated enhanced performance across nearly all tests, with the exception of SCWT completion time. Groups that incorporated cognitive elements into their warm-up showed improvements in inhibition and accuracy, as reflected by the significantly lower error rates in the Stroop test. These results suggest an enhanced ability to process conflicting stimuli and suppress automatic responses—skills attributed to executive control and attentional focus [29]. The superior outcomes of the motor–cognitive groups may stem from the increased mental engagement and focus required to respond to simultaneous stimuli during warm-up. The SCWT, a classical measure of inhibition and cognitive control, revealed that only the error rate—not the total time—differed between groups. This aligns with previous findings suggesting that combined cognitive–physical protocols may enhance accuracy but not necessarily reaction time [7]. Performance in IBT, a complex agility task involving ball control, direction changes, and spatial awareness, also reflected cognitive benefits. MVEG and MVIG outperformed MG in completion time, likely due to improvements in multi-tasking and attentional control—key components in performing task sequences and executing precise motor actions. Previous research has shown that motor–cognitive interventions can lead to immediate improvements in working memory. Our findings may further support the notion that ball-dribbling agility tasks engage not only physical capacities but also cognitive processes. This dual demand has been documented in earlier studies, reinforcing the view that such tasks reflect the complex interplay between motor execution and cognitive control [5,23,26,27]. However, in the present study, these cognitive processes were only indirectly involved and were not directly assessed. Consequently, although the IBT outcomes may suggest cognitive benefits, alternative factors—such as heightened arousal or increased motivation—cannot be excluded as potential explanations. In the LSPT, which demands both football-specific skills and executive functions such as decision-making, attentional control, and reaction speed, MVEG and MVIG again performed significantly better than MG. Previous research has characterized the LSPT as cognitively demanding, requiring players to respond to randomized stimuli and make rapid decisions under time constraints [24]. In the present study, while such executive functions may have played a role, their influence cannot be isolated with certainty. Other performance-related factors—such as heightened physiological activation or task-related engagement—may have also contributed to the observed outcomes. These results underscore the relevance of warm-up tasks that challenge the athlete’s ability to process real-time sensory input, interpret signals, and adjust movements accordingly. Enhanced shifting ability—a domain of executive functioning associated with dynamic task switching—may have contributed to the observed performance gains [5]. Interestingly, while MVIG achieved the best results in the LSPT, their performance declined in the *t*-test, suggesting potential drawbacks of visual processing in more reactive or speed-dependent tasks. Prior evidence suggests that visual stimuli, while informative, require more cognitive resources and may lead to greater neural fatigue when overexposed [30,31]. Visual exploration demands more effort than auditory processing, as 83% of environmental data is processed visually compared to 11% auditorily [15]. Moreover, visual signals are generally processed more slowly than auditory ones [32], which could partially explain the *t*-test results favoring the motor–verbal group. Additionally, it is plausible that participants in the motor–visual group experienced cognitive overload or fatigue during testing due to the high visual demands of their warm-up routine. Studies have shown that prolonged exposure to visual stimuli—particularly in combination with task demands—can temporarily reduce response accuracy and speed [31,33]. In contrast, the MVEG protocol may have reduced mental effort more efficiently. Auditory cues, processed faster and with less perceptual strain, might have facilitated quicker decision-making and better integration of motor responses. These findings support the utility of motor–cognitive warm-ups involving auditory instructions to enhance short-term executive and motor functioning in young athletes. Similar cross-modal effects have been observed, where auditory stimulation accelerates visual response times [34].

In the *t*-test, the group with integrated auditory stimuli excelled, completing the test fastest, while the motor-only group was quicker than the motor–visual group. This suggests that motor–cognitive exercises involving auditory cues may be more effective for enhancing short-term agility performance. The superior performance of MVEG in this test, which requires rapid direction changes and coordination, could be attributed to the lower cognitive load of auditory processing. One plausible explanation is that auditory-based warm-ups may have imposed less cognitive and motor strain on participants compared to visual ones, potentially allowing for more efficient allocation of attentional resources. This interpretation aligns with previous findings suggesting that auditory cues are typically processed more rapidly and with lower cognitive demand than visual signals [32]. Although we did not directly assess cognitive load or fatigue, this remains a plausible—though unconfirmed—mechanism. Additionally, the observed group differences in *t*-test performance may reflect not only cognitive factors but also broader influences such as individual responsiveness or task familiarity. Notably, auditory cue-based motor–cognitive tasks may have supported more streamlined sequencing and faster directional transitions, contributing to quicker response execution and more coordinated movement—skills, particularly relevant to the demands of the *t*-test.

Collectively, our findings underscore that integrating sensory-based cognitive stimuli into warm-ups can acutely enhance both cognitive and motor abilities in young football players. However, stimulus type should be tailored to the specific training objectives. The present findings also provide guidance for coaches designing warm-ups. Motor–visual protocols may be especially useful when training goals emphasize passing accuracy, visual scanning, and decision-making under pressure. In contrast, motor–verbal protocols may be better suited for sessions targeting agility, rapid direction changes, or dribbling skills, as they seem to place less perceptual load on players. Coaches may therefore select the type of cognitive stimulus depending on the specific technical or physical focus of the session.

We focused on the short-term effects through standardized tests rather than match performance; the results are still relevant to game preparation. The selected tests (Stroop, LSPT, IBT, and *t*-test) reflect abilities that are highly important in match play, such as decision-making under pressure, attentional control, dribbling with quick direction changes, and passing and agility with and without the ball. It is noteworthy that we did not record the participants’ performance during actual matches, so we cannot directly confirm that these warm-ups enhanced in-game outcomes. However, by integrating cognitive elements, coaches may simultaneously stimulate both technical and cognitive skills of the players.

This study presents several limitations. The relatively small sample size, drawn from a single club and age category, reduces statistical power and limits the generalizability of the findings to broader populations. Furthermore, cognitive load was not experimentally manipulated, which restricts the ability to attribute performance differences exclusively to executive functions. It is possible that other factors—such as arousal, motivation, or task engagement—also contributed to the observed outcomes. Pre-tests and baseline assessments were intentionally omitted to prevent participants from becoming overly familiar with the tests prior to the intervention. Although randomization and group similarity in age, anthropometric characteristics, and training background likely minimized bias, baseline differences cannot be entirely ruled out.

Future research should explore how different sensory modalities—such as auditory and visual cues—in motor–cognitive warm-up protocols affect cognitive and motor performance across various age groups and in female athletes. Investigating how these stimulus-based interventions influence performance under different game-relevant conditions could offer deeper insight into individual responsiveness. Tailoring warm-up strategies to athletes’ developmental stages and sensory preferences may enhance their effectiveness and support more comprehensive training approaches in youth football programs. Further investigation is warranted to explore the effects of motor–cognitive warm-up protocols on actual match performance. Beyond test outcomes, future research should examine whether such warm-ups enhance technical–tactical execution and decision-making during real-game situations.

## 5. Conclusions

This study demonstrates that incorporating motor–cognitive warm-up routines based on auditory or visual stimuli might lead to immediate improvements in both cognitive and motor performance among youth football players. Participants exposed to verbal (auditory) or visual cues performed significantly better in tasks assessing executive functions, agility, and decision-making compared to those engaging in motor-only warm-ups.

Warm-ups involving auditory cues appeared particularly effective for enhancing agility-related performance (e.g., *t*-test, IBT), whereas visual-stimulus-based protocols proved advantageous in tasks requiring visual scanning and passing precision (e.g., LSPT). These effects were observed immediately following the warm-up and within controlled testing conditions, rather than during actual competitive play. Therefore, while the results cannot be directly generalized to match performance, they nonetheless suggest that tailoring warm-up protocols to specific training objectives may enhance both cognitive and motor preparedness in young athletes. The insights gained from this study may prove valuable for coaches and trainers seeking to simultaneously develop motor and cognitive performance in their players.

## Figures and Tables

**Table 1:** Anthropometric data and training experience of the participants (mean ± standard deviation).

Variables	MVEG (*n* = 8)	MVIG (*n* = 8)	MG (*n* = 8)	*p*-Value
Age (year)	8.42 ± 0.17	8.68 ± 0.20	8.56 ± 0.33	
Weight (kg)	26.89 ± 2.56	31.09 ± 6.03	32.26 ± 3.65	0.082
Height (cm)	131.19 ± 4.49	133.13 ± 3.56	135.00 ± 5.50	0.271
BMI	15.79 ± 0.88	17.32 ± 2.59	17.46 ± 0.90	0.060
Training experience in the same team (year)	2 ± 0	2 ± 0	2 ± 0	

BMI = body mass index, MVEG = motor–verbal group, MVIG = motor–visual group, MG = motor-only group, *p* ≤ 0.05.

## Data Availability

Data is not available due to privacy restrictions. The data presented in this study are available on request from the corresponding author.
